# Increased serum thrombomodulin level is associated with disease severity and mortality in pediatric sepsis

**DOI:** 10.1371/journal.pone.0182324

**Published:** 2017-08-03

**Authors:** Jainn-Jim Lin, Hsiang-Ju Hsiao, Oi-Wa Chan, Yu Wang, Shao-Hsuan Hsia, Cheng-Hsun Chiu

**Affiliations:** 1 Division of Pediatric Critical Care and Pediatric Sepsis Study Group, Chang Gung Children’s Hospital and Chang Gung Memorial Hospital, Chang Gung University College of Medicine, Taoyuan, Taiwan; 2 Graduate Institute of Clinical Medical Sciences, College of Medicine, Chang Gung University, Taoyuan, Taiwan; 3 Molecular Infectious Disease Research Center, Chang Gung Children’s Hospital and Chang Gung Memorial Hospital, Chang Gung University College of Medicine, Taoyuan, Taiwan; 4 Department of Pediatrics, Chang Gung Memorial Hospital at Keelung, College of Medicine, Chang Gung University, Taoyuan, Taiwan; 5 Department of Pediatrics, Chang Gung Memorial Hospital, Chiayi, Taiwan; Royal College of Surgeons in Ireland, IRELAND

## Abstract

**Background:**

Endothelial dysfunction plays an important role in the pathophysiology of sepsis. As previously reported, the serum thrombomodulin is elevated in diseases associated with endothelial injury.

**Objective:**

The aim of this study was to investigate the association of serum thrombomodulin level in different pediatric sepsis syndromes and evaluate the relationship with disease severity and mortality.

**Methods:**

We prospectively collected cases of sepsis treated in a pediatric intensive care unit from June 2012 to July 2015 at Chang Gung Children’s Hospital in Taoyuan, Taiwan. Clinical characteristics and serum thrombomodulin levels were analyzed.

**Results:**

Increased serum thrombomodulin levels on days 1 and 3 of the diagnosis of sepsis were found in different pediatric sepsis syndromes. Patients with septic shock had significantly increased serum thrombomodulin levels on days 1 and 3 [day 1: median, 6.9 mU/ml (interquartile range (IQR): 5.8–12.8) and day 3: median, 5.8 mU/ml (IQR: 4.6–10.8)] compared to healthy controls [median, 3.4 mU/ml (IQR: 2.3–4.2)] (p = <0.001 and 0.001, respectively) and those with sepsis [day 1: median, 2.9 mU/ml (IQR: 1.8–4.7) and day 3: median, 3 mU/ml (IQR: 1.5–3.5)] and severe sepsis [day 1: median, 3.3 mU/ml (IQR: 1.3–8.6) and day 3: median, 4.4 mU/ml (IQR: 0.5–6)] (p = <0.001 and 0.001, respectively). There was also a significant positive correlation between serum thrombomodulin level on day 1 and day 1 PRISM-II, PELOD, P-MOD and DIC scores. The patients who died had significantly higher serum thrombomodulin levels on days 1 and 3 [day 1: median, 9.9 mU/ml (IQR: 6.2–15.6) and day 3: median, 10.4 mU/ml (IQR: 9.2–11.7)] than the survivors [day 1; median, 4.4 mU/ml (IQR: 2.2–7.5) and day 3: [median, 3.5 mU/ml (IQR: 1.6–5.7)] (p = 0.046 and 0.012, respectively).

**Conclusion:**

Increased serum thrombomodulin levels were found in different pediatric sepsis syndromes and correlated with disease severity and mortality.

## Introduction

Treating sepsis is a challenge, and it is one of the leading causes of admissions to pediatric intensive care units. The initial manifestation can often be nonspecific and misleading. Severe sepsis is frequently complicated by disseminated intravascular coagulation (DIC) and multiple organ dysfunction syndromes (MODS). Deterioration from sepsis to septic shock and MODS most often occurs in the first 24 hours [1]. In our previous study, we found decreased a disintegrin and metalloprotease with a thrombospondin type 1 motif, member 13 (ADAMTS 13) activity is associated with disease severity and outcome in pediatric severe sepsis [2].Despite an improved understanding of the pathophysiology of sepsis, the overall mortality rate remains unacceptably high [3].

Endothelial dysfunction plays an important role in the pathophysiology of sepsis. Thrombomodulin, a membrane glycoprotein, is expressed on vascular endothelial cells and is found in the body in a bound form and a soluble or plasmatic form [4]. It binds to thrombin and acts as an anticoagulant. Once thrombomodulin binds to endothelial thrombin, the thrombin-thrombomodulin complex accelerates protein C activation [4, 5], and soluble thrombomodulin is released from the surface of endothelial cells into the serum by proteolytic degradation [6]. As previously reported, the serum thrombomodulin is elevated in diseases associated with endothelial injury, such as acute respiratory distress syndrome [7,8], disseminated intravascular coagulation [9], and organ dysfunction induced by sepsis [10–12]. Therefore, thrombomodulin is known to be a biomarker of endothelial injury.

Since endothelial dysfunction plays an essential role in the pathogenesis of sepsis, some studies have focused on serum thrombomodulin level as a predictor of the severity of sepsis and mortality in adults [10–12]. However, few pediatric studies have examined the role of serum thrombomodulin as a biomarker to predict the clinical course of different pediatric sepsis syndromes, sepsis-induced DIC, MODS and mortality [13]. Besides, increased serum thrombomodulin levels have also been reported in conditions other than sepsis that cause ischemic and/or inflammatory endothelial injuries [7–9, 14–15]. The aim of this study was to investigate the association of serum thrombomodulin level in different pediatric sepsis syndromes, and to evaluate the relationship with disease severity and mortality.

## Materials and methods

### Design, patients and intensive care unit setting

This was a prospective study conducted over 36 months in a 29-bed pediatric intensive care unit (PICU) at Chang Gung Children’s hospital from July 2012 to June 2015. Previously healthy children diagnosed with sepsis on the first day of admission to the PICU were included. The patients were divided into three groups according to the guidelines of the Society of Critical Care Medicine Consensus Conference Committee as sepsis, severe sepsis and septic shock [16]. In addition, organ failure was categorized according to the same guidelines as respiratory, cardiovascular, central nervous system failure, hepatic, renal, hematologic and metabolic failure [16]. Multiple organ dysfunction syndrome (MODS) was defined as the dysfunction of more than two organs. The DIC score was based on International Society on Thrombosis and Haemostasis subcommittee guidelines, with DIC being defined as a score of 5 or more [17]. The presence of different sepsis syndrome, DIC and MODS was determined on day 1 of sepsis. The patients with any known underlying diseases such as hematological disorders or liver disease were excluded. Therefore, we limited our study patients to previously healthy children and excluded any possible etiology of endothelial injury. Controls were recruited from 15 healthy children age 5–8 years in the outpatient clinic for tic disorders.

The study was approved by the Ethical Review Committee of Chang Gung Memorial Hospital (Approval IRB Number: 100-2984A, 103-3076C, 103-4188C and 104-3052C). Written informed consent was obtained from the participants or their parents before blood collection

The following information was collected: age, gender, site of infection, laboratory findings, mechanical ventilation requirement, vasopressor use, and severity of disease as assessed on day 1 of blood sampling according to the Pediatric Risk of Mortality (PRISM) III, Pediatric Logistic Organ Dysfunction (PELOD), Pediatric-Multiple Organ Dysfunction (P-MOD), and disseminated intravascular coagulation (DIC) scores.

### Blood sampling and measurement

Blood samples were collected on days 1 and 3 of admission to the PICU. Day 3 samples were collected for survivors by day 3. Serum was obtained after centrifugation and the samples were labeled and kept at -80°C until analysis. Serum soluble thrombomodulin was quantified using an IMUBIND Thrombomodulin ELISA kit (Sekisui Diagnostics, Lexington, MA, USA), according to the manufacturer’s instructions.

### Statistical analysis

Data are presented as median and interquartile range (IQR, 25th-75th percentile) or number (%). Since most continuous variables were skewed, nonparametric approaches were used in this study. The Mann-Whitney U test was used for continuous and ordinal variables, and the chi-square test was used for nominal and categorical variables depending on the scale of measurement. The Kruskal-Wallis test was used for test for differences when comparing more than two groups. Spearman’s rank correlation coefficients were determined to evaluate correlations between serum thrombomodulin level and severity score and other laboratory parameters.

Differences in serum thrombomodulin level between different pediatric sepsis syndromes and the controls were analyzed. Serum thrombomodulin levels were also compared between patients with and without DIC and with and without MODS, and between survivors and non-survivors. Receiver operating characteristic (ROC) curves for day 1 serum thrombomodulin level to predict the development of septic shock, DIC, MODS, and mortality were plotted. The respective areas under the ROC curves and cut-off points were calculated. Statistical analysis was performed using SPSS statistical software, version 17.0 (SPSS, Inc., Chicago, IL, U.S.A.). A p value of less than 0.05 was considered to be statistically significant. All statistical tests were two-tailed.

## Results

### Demographic characteristics

Forty-two previously healthy children with sepsis included 24 (57.1%) males and 18 (42.9%) females, of whom 14 (33.3%) had sepsis, 13 (31%) had severe sepsis, and 15 (35.7%) had septic shock. The median age of the children was 4.1 years (IQR range 1.7–8.7). Microbiologically defined infections were found in 22 (52.4%) patients, including *Streptococcus pneumonia* in 18 (42.9%), *Salmonella group B* or *D* in two (4.7%), *Escherichia coli* in one (2.4%), and *Mycoplasma pneumonia* in one (2.4%). The most frequent infection sites were the respiratory system (n = 18, 42.9%), central nervous system (n = 6, 14.3%), and the heart (n = 4, 9.5%).

Nineteen (45.2%) patients needed mechanical ventilator support, and 15(35.7%) received inotropic agents. There were significant differences in the disease severity at admission among the different sepsis syndrome as assessed by PRISM-III, PELOD, P-MODS and DIC scores (Table 1). The durations of ICU stay and hospitalization were 5.5 (3–9) and 15 (9–21) days, respectively, in the sepsis group, 9 (7–15) and 16 (13–32) days, respectively, in the severe sepsis group, and 9 (4–16) and 16 (11–29) days, respectively, in the septic shock group. However, the difference was not statistically significant. The demographic data of different pediatric sepsis syndromes are listed in Table 1.

**Table 1:** Demographic data of patients with different sepsis syndromes (n = 42).

Characteristic	Sepsis (n = 14)	Severe sepsis (n = 13)	Septic shock (n = 15)	*p* value
Gender (Male/Female)	8/6	7/6	9/6	0.948
Age	4.9 (2.1–10.1)	1.7 (0.2–3.6)	6.8 (4.5–14.6)	0.067
**Site of infection**				-
Respiratory	7	6	5	
Central nervous system	3	2	1	
Cardiovascular	1	0	3	
Digestive	2	0	0	
Urinary	0	1	1	
Other/unknown	1	4	5	
**Laboratory**				
Defined pathogen	9	6	7	0.550
**Organisms**				-
*Streptococcus pneumoniae*	7	6	5	
*Salmonella* group B or D	2	0	0	
*Escherichia coli*	0	0	1	
*Mycoplasma pneumoniae*	0	0	1	
White blood count (10^3^//μL)	8.6 (5.5–12.9)	7.3 (3.8–13.1)	8.7 (2.6–12.5)	0.969
Hemoglobulin (mg/dL)	13.1 (10.7–14.6)	10.3 (9.1–11)	12.2 (8.5–12.8)	0.024*
Platelet (10^3^/μL)	209 (143–246)	175 (38.5–410)	161 (40–224)	0.147
Thrombomodulin (D1) (mU/ml)	2.9 (1.8–4.7)	3.3 (1.3–8.6)	6.9 (5.8–12.8)	0.001*
Thrombomodulin (D3) (mU/ml)	3 (1.5–3.5)	4.4 (0.5–6)	5.8 (4.6–10.8)	0.002*
**Disease severity**				
Mechanical ventilation	0	4	15	<0.001*
Use of vasopressor	0	0	15	<0.001*
PRISM-III score	7 (5–9)	13 (9–16)	22 (19–45)	<0.001*
PELOD score	10 (0–11)	11 (2–12)	31 (22–50)	<0.001*
P-MODS	0 (0–1)	1 (0–3)	6 (3–9)	<0.001*
DIC score	2 (0–2)	3 (1–3)	5 (3–5)	<0.001*
**Outcome**				
Mortality	0	0	4	0.019*
ICU stay (days)	5.5 (3–9)	9 (7–15)	9 (4–16)	0.148
Hospitalization (days)	15 (9–21)	16 (13–32)	16 (11–29)	0.663

Abbreviations: D1, day 1; D3, day 3; PRISM, Pediatric Risk of Mortality; PELOD, Pediatric Logistic Organ Dysfunction; P-MODS, Pediatric-Multiple Organ Dysfunction Score; DIC, disseminated intravascular coagulation; ICU, intensive care unit

*p<0.05, statistically significant

### MODS and DIC

Fifteen (35.7%) patients had MODS and 10 (23.8%) patients had DIC on day 1. There were no differences between the patients with and without MODS and DIC in age and gender. The patients with MODS and DIC had higher disease severity as indicated by PRISM-III, PELOD, P-MODS and DIC scores than those without (Table 2). The overall PICU mortality rate was 9.5%, and it was higher in the patients who had MODS and DIC than in those who did not (mortality rate 26.7% in those with MODS and 40% in those with DIC, p = 0.012 and 0.002, respectively). The patients who had MODS and DIC had an up to 6-fold and 3.5-fold higher mortality rate than those who did not, respectively.

**Table 2:** Clinical characteristics of patients with disseminated intravascular coagulation, multiple organ dysfunction syndrome and mortality.

Characteristic	DIC			MODS			Mortality		
	Present (n = 10)	Absent (n = 32)	P value	Present (n = 15)	Absent (n = 27)	P value	Non-survivors (n = 4)	Survivors (n = 38)	P value
Age (years)	4.8 (2.9–7.2)	3.6 (1.2–9.6)	0.991	4.9 (1.8–9.6)	3.4 (0.8–7)	0.121	5.7 (1.4–8)	3.9 (1.7–9.5)	0.818
Gender (M/F)	5/5	19/13	0.601	9/6	15/12	0.780	2/2	22/16	1.000
**Disease severity**									
PRISM-III score	34 (21–48)	9 (7–16)	<0.001*	22 (18–45)	9 (6–13)	<0.001*	48 (39–52)	11 (7–19)	<0.001*
PELOD score	42 (28–51)	11 (1–20)	<0.001*	31 (20–50)	11 (1–12)	<0.001*	50 (36–59)	12 (2–20)	<0.001*
P-MODS	7 (5–9)	1 (0–2)	<0.001*	6 (4–9)	1 (0–1)	<0.001*	9 (7–9)	1 (0–4)	<0.001*
DIC score	5 (5–6)	2 (1–3)	<0.001*	5 (2–5)	2 (1–3)	<0.001*	5 (5–7)	2 (2–3)	0.026*
**Serum thrombomodulin level**									
TM (D1) (mU/ml)	8.3 (6.3–14.8)	3.6 (1.9–5.7)	0.010*	8.7 (6–13.4)	3 (1.4–5.3)	0.013*	9.9 (6.2–15.6)	4.4 (2.2–7.5)	0.046*
TM (D3) (mU/ml)	5.9 (5–11.7)	3.5 (1.6–5.4)	0.028*	5.9 (4.5–11.1)	3.3 (1.5–5)	0.017*	10.4 (9.2–11.7)	3.5 (1.6–5.7)	0.018*
**Outcome**									
Mortality (%)	4 (40%)	0	0.002*	4 (26.7%)	0	0.012*	-	-	-

Abbreviations: M, male; F, female; TM, thrombomodulin; D1, day 1; D3, day 3; PRISM, Pediatric Risk of Mortality; PELOD, Pediatric Logistic Organ Dysfunction; MODS, Multiple Organ Dysfunction Syndrome; P-MODS, Pediatric-Multiple Organ Dysfunction Score; DIC, disseminated intravascular coagulation; NS, not significant (*p*>0.05)

*p<0.05, statistically significant

### Serum thrombomodulin level and disease severity

Increased serum thrombomodulin levels on days 1 and 3 of admission were found in different pediatric sepsis syndromes. The serum thrombomodulin levels on days 1 and 3 were significantly higher in the septic shock group than in the sepsis and severe sepsis groups (p = 0.001 and 0.002, respectively) (Table 1). The patients with septic shock syndromes had significant increased serum thrombomodulin levels on days 1 and 3 of admission compared to the 15 healthy controls [median, 3.4 mU/ml (IQR: 2.3–4.2), p = <0.001 and 0.001, respectively]. In addition, the serum thrombomodulin levels on days 1 and 3 were significantly higher in the patients with MODS and DIC than in those without (Table 2). There were also a strong positive correlations between serum thrombomodulin level on day 1 and baseline PRISM-III, PELOD, P-MODS, and DIC scores, but we addressed the problem with multiple hypothesis testing and no adjustment of the p value for this. The correlations of serum thrombomodulin levels and disease severity are summarized in Table 3.

**Table 3:** Correlations between serum thrombomodulin levels on day 1 and day 1 disease severity and laboratory parameters.

Characteristic	Correlation coefficients	P value
**Clinical parameter**		
PRISM-III	0.543	<0.001*
PELODs	0.719	<0.001*
P-MOS	0.571	<0.001*
**Laboratory parameter**		
Platelet	-0.307	0.057
PT	0.469	0.003*
Fibrinogen	-0.289	0.079
DIC score	0.553	<0.001*
Blood urea nitrogen (BUN)	0.386	0.015*
Creatinine (Cr)	0.330	0.040*

Abbreviations: D1, day 1; PRISM, Pediatric Risk of Mortality; PELOD, Pediatric Logistic Organ Dysfunction; MODS, Multiple Organ Dysfunction Syndrome; P-MODS, Pediatric-Multiple Organ Dysfunction Score; DIC, disseminated intravascular coagulation

*P < 0.05, statistically significant

### Serum thrombomodulin level and mortality

In-hospital mortality occurred in four (9.5%) patients, all of whom had septic shock. The mortality rate was significantly higher in the septic shock group compared to the sepsis and severe sepsis groups (*p* = 0.019). One patient died prior to day 3 and excluded to analysis the relationship between day 3 TM and mortality. The serum thrombomodulin levels on days 1 and day 3 of admission in the non-survivors were significantly higher than in the survivors (*p* = 0.046 and 0.012, respectively) (Table 2).

### Area under the ROC curve for day 1 serum thrombomodulin levels in predicting septic shock, DIC, MODS and mortality

**Fig 1:**
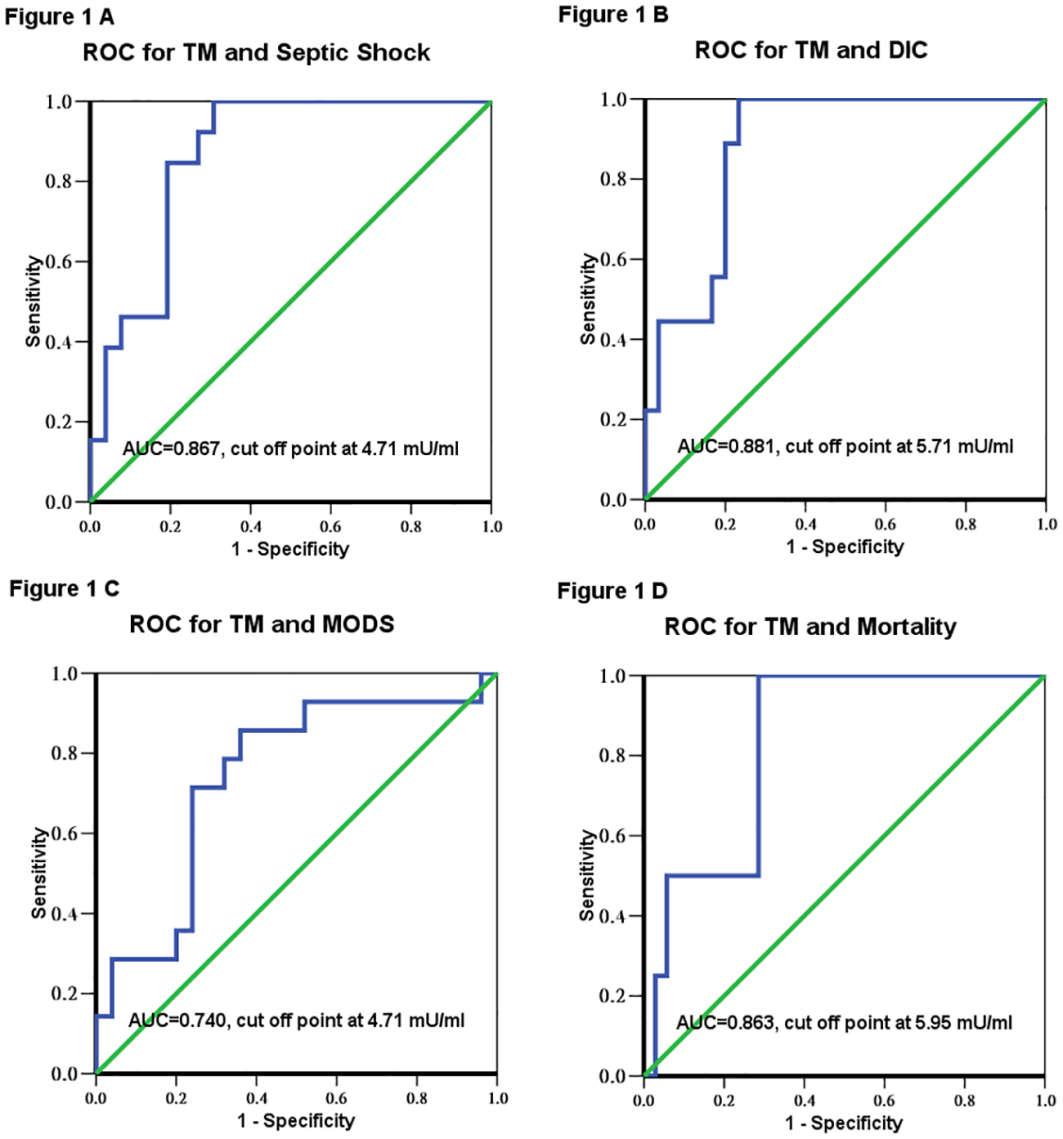
Receiver operating characteristic (ROC) curves and the area under the ROC curves (AUC) and cut-off points for serum thrombomodulin level. The definition of different endpoints were seen in the text. The areas under the ROC curves and cut-off points showed that day 1 serum thrombomodulin levels had good discriminative power in predicting the development of (A) septic shock (AUC = 0.867, cut-off point 4.71 mU/ml), (B) disseminated intravascular coagulation (DIC) (AUC = 0.881, cut-off point 5.71 mU/ml), (C) multiple organ dysfunction syndrome (MODS) (AUC = 0.740, cut-off point 4.71 mU/ml), and (D) mortality (AUC = 0.863, cut-off point 5.95 mU/ml).

Areas under the ROC curves for serum thrombomodulin levels on day 1 of sepsis in predicting septic shock, DIC, MODS and mortality are shown in Fig 1. The areas under the ROC curves and cut-off points showed that day 1 serum thrombomodulin levels had good discriminative power in predicting the development of septic shock (AUC = 0.867, cut-off point 4.71 mU/ml), DIC (AUC = 0.881, cut-off point 5.71 mU/ml), MODS (AUC = 0.740, cut-off point 4.71 mU/ml), and mortality (AUC = 0.863, cut-off point 5.95 mU/ml) (Fig 1).

## Discussion

Biomarkers of endothelial dysfunction are not recommended for routine laboratory investigations in pediatric sepsis, and few studies have examined the role of serum thrombomodulin as a biomarker to predict the clinical course of different sepsis syndromes, sepsis-induced DIC, MODS and mortality in children. In this study, we demonstrated increased serum thrombomodulin levels in children with different sepsis syndromes, sepsis-induced disseminated intravenous coagulopathy and multiple organ failure syndromes. The serum thrombomodulin level was also strongly positively correlated with disease severity of pediatric sepsis. In addition, increased serum thrombomodulin levels on days 1 and 3 after the diagnosis of sepsis were also associated with an increased risk of mortality. Analysis of areas under the ROC curves confirmed that day 1 serum thrombomodulin levels had good discriminative power in predicting the development of septic shock, DIC, MODS and mortality.

Increased serum thrombomodulin levels have been reported in different sepsis syndromes and to be correlated with disease severity. Mihajlovic et al conducted a 1-year prospective study in 60 adults with sepsis and found that the serum thrombomodulin level could be used to predict the severity of sepsis, and also that it was a significant predictor of the development of MODS [11]. Lin et al conducted a prospective study in 100 adults with sepsis, and also found that serum thrombomodulin level independently predicted the development of disseminated intravascular coagulation (hazard ratio 1.13) and multiple organ dysfunction syndrome (hazard ratio 1.12) [10]. In addition, Krafte-Jacobs et al conducted a 2-year prospective study in 22 children with septic shock, and found a higher serum mean thrombomodulin level with an increasing number of organ failures [13]. In the current study, we found higher serum thrombomodulin levels on days 1 and 3 in the patients with different sepsis syndromes. There were also significant positive correlations between serum thrombomodulin levels on days 1 and day 1 disease severity scores. Therefore, measurements of serum thrombomodulin still help to early recognize the development of septic shock, sepsis-induced DIC and MODS.

Serum thrombomodulin levels may also be used to monitor the evolution of DIC and MODS in patients with sepsis. Lin et al reported that the serum thrombomodulin level decreased in patients with resolved DIC and MODS, while they remained high in those with persistent DIC and MODS. These findings suggest that endothelial injury plays a role in progression from sepsis to DIC and MODS, and subsequently mortality [10]. Therefore, the serum thrombomodulin level in patients with sepsis may be used as a biomarker to monitor the resolution or aggravation of DIC and MODS.

Serum thrombomodulin levels may also be used as an early predictor of mortality in pediatric patients with sepsis. Lin et al reported that the serum thrombomodulin level predicted mortality (hazard ratio 1.19) in adults with sepsis [10], and Krafte-Jacobs et al reported 1.5- and 3-fold higher serum thrombomodulin levels in pediatric patents who survived and died from septic shock, respectively, compared to healthy controls [13]. In our study, we also found that the pediatric patients who died had significantly higher serum thrombomodulin levels on days 1 and 3 than the survivors.

## Limitations

There are several limitations to this study. First, this is a small prospective study which included only 42 patients. Besides, there were only less than 20 patients in the septic shock group. So, it is difficult to make a firm recommendation for the benefit of serum thrombomodulin. Further studies with a larger sample size are needed to confirm our results. Second, disease severity scores were only analyzed on day 1 after the diagnosis of sepsis. As analysis of long-term serial scores was not performed, we could not evaluate dynamic changes in serum thrombomodulin level with the development of sepsis. This would have been interesting to evaluate potential therapeutic strategies.

Third, we only determined serum thrombomodulin levels and we did not analyze other biomarkers of coagulopathy or vascular injury such as protein C, protein S [11], ICAM-1, VCAM-1 and E-selectin [10]. Therefore, it is difficult to confirm the pathophysiological role of thrombomodulin in sepsis. Finally, the 15 controls were from the outpatient clinic for tic disorders. Whether the serum thrombomodulin level was normal in this group is unknown. Age- and gender-matched healthy controls may have been more appropriate.

## Conclusion

This study demonstrated that serum thrombomodulin level, a biomarker of endothelial injury, was increased in different pediatric sepsis syndromes and correlated with disease severity and mortality. Serum thrombomodulin level may be a useful tool in disease severity and mortality. Measurements of serum thrombomodulin may help to early recognize the development of septic shock, sepsis-induced DIC and MODS to allow for more appropriate therapeutic strategies.

**Key messages**

1. Increased serum thrombomodulin levels were found in different pediatric sepsis syndromes and correlated with disease severity and mortality.
2. Measurements of serum thrombomodulin may help to early recognize the development of septic shock, sepsis-induced DIC and MODS to allow for more appropriate therapeutic strategies.

## Abbreviations

DIC: disseminated intravascular coagulation
ELISA: enzyme-linked immunosorbent assay
PELOD: Pediatric Logistic Organ Dysfunction
PICU: pediatric intensive care unit
P-MOD: Pediatric-Multiple Organ Dysfunction
PRISM: Pediatric Risk of Mortality
